# Investigation of Endogenous Renal CEST Contrast and the Influence of Respiratory Motion on a Clinical 3 Tesla MRI: An In Vivo and In Vitro Study

**DOI:** 10.1002/mrm.70210

**Published:** 2025-12-03

**Authors:** Patrik Jan Gallinnis, Benedikt Kamp, Karl Ludger Radke, Rika Möller, Anna‐Katharina Juric, Julia Stabinska, Vít Herynek, Gerald Antoch, Hans‐Jörg Wittsack, Alexandra Ljimani, Anja Müller‐Lutz

**Affiliations:** ^1^ Medical Faculty, Department of Diagnostic and Interventional Radiology University Dusseldorf Dusseldorf Germany; ^2^ F.M. Kirby Research Center for Functional Brain Imaging Kennedy Krieger Institute Baltimore Maryland USA; ^3^ Russell H. Morgan Department of Radiology and Radiological Science Johns Hopkins University School of Medicine Baltimore Maryland USA; ^4^ Center for Advanced Preclinical Imaging (CAPI), First Faculty of Medicine Charles University Prague Czech Republic; ^5^ CARID, Cardiovascular Research Institute Düsseldorf University Hospital Düsseldorf, Heinrich‐Heine‐University Dusseldorf Germany

**Keywords:** abdominal CEST, amide proton transfer, chemical exchange saturation transfer, dynamic phantom, kidney, magnetization transfer imaging

## Abstract

**Purpose:**

The aim is to evaluate the effectiveness of timed breathing in reducing respiratory motion artifacts in renal chemical exchange saturation transfer (CEST) MRI and to assess potential differences in CEST effects between renal compartments.

**Methods:**

An electro‐pneumatic phantom with a kidney CEST model simulated variable respiratory motion and sequence‐synchronized breathing. Motion‐induced deviations from a static reference were quantified using the mean absolute error (MAE). Ten healthy volunteers (six females, four males; 25.2 ± 1.9 years) and one patient (47 years) with ccRCC (3.0 × 2.2 × 2.2) cm^3^ were examined on a 3 T MRI system using a multi‐echo gradient echo sequence with 15 Gaussian‐shaped saturation pulses (B_1_ = 1.5 μT, *t*_p_ = *t*_ipd_ = 100 ms). CEST effects in cortex, medulla, and pelvis at 1.0, 2.0, and 3.5 ppm were quantified by MTR_asym_ analysis under timed and free‐breathing conditions.

**Results:**

Timed breathing reduced motion artifacts in both phantom and in vivo data. MTR_asym_ analysis exhibits visibly distinguishable and significantly different (*p* < 0.05) CEST effects in renal compartments, with increased MTR_asym_ values of (0.78% ± 0.41%) at 1.0 ppm in the cortex, (1.43% ± 0.70%) at 2.0 ppm in the medulla, and (−2.02% ± 0.84%) at 3.5 ppm in the pelvis. Significant differences (*p* < 0.05) in MTR_asym_ values were observed between the patient and the healthy cohort.

**Conclusion:**

Timed breathing improves renal CEST MRI by reducing motion artifacts and enabling detection of compartment‐specific CEST effects, highlighting its potential for biochemical characterization of renal tissue in clinical applications.

## 1 Introduction

Functional magnetic resonance imaging (fMRI) is increasingly used in the medical assessment of the kidney as it can detect pathophysiological changes and visualize clinically relevant processes that cannot be detected by morphological imaging techniques [1]. Chemical exchange saturation transfer (CEST) imaging represents a novel MRI method in the field of renal imaging and has the potential to complement established methods with metabolic information in clinical diagnostics [2, 3].

CEST imaging is based on the exchange of protons between water molecules and specific metabolites, proteins, or peptides [4]. This technique is of particular interest in the field of renal imaging, as the functionally distinct compartments of the kidney are associated with a range of metabolic processes. In the cortex, blood is filtered through ultrafiltration, and the resulting ultrafiltrate is concentrated in the proximal tubules of the medulla through reabsorption, before being excreted via the renal pelvis [5]. Metabolites such as urea, glucose, creatine and creatinine play key roles in these processes, and their proton exchange with water can be detected using CEST MRI [6].

CEST‐MRI uses frequency‐selective saturation pulses to saturate protons in certain metabolites that reduce the signal of the water after proton exchange [4]. This frequency‐dependent signal reduction becomes visible in a Z‐spectrum and allows the indirect detection of molecular groups.

Glucose, urea, and citric acid typically exhibit exchange effects around 1.0 ppm relative to the water resonance [6]. Glucose has another exchangeable proton bound that can be excited together with creatine at about 2.0 ppm [6]. Metabolites containing amide groups can be detected around 3.5 ppm [6, 7]. These amide protons are of particular interest due to their increased contribution in tumors and their pH‐sensitive exchange rates [8, 9].

Preclinical studies suggest that renal pH changes, may serve as early indicators of chronic kidney disease (CKD) [10], renal obstruction [11], and acute kidney injury [12]. Since the kidney plays a central role in maintaining the body's acid‐base balance [13], pH mapping via amide proton transfer (APT)‐CEST could offer valuable insights into renal function. Furthermore, histological findings by Tostain et al. [14] on clear cell renal carcinomas showed an increased expression of proteins that enable a neutral pH value in the acidic microenvironment of the tumor and thus promote the proliferation, migration, and invasion of cancer cells. These findings point to the potential of renal APT‐CEST imaging for assessing kidney health and tumor classification [15] or tumor activity.

However, these potential clinical applications of renal CEST imaging are associated with challenges [3]. A central problem in MRI examinations of native kidneys remains motion caused by respiration. Primarily, respiratory motions in the superior–inferior direction affect the accuracy of the acquired Z‐spectra, as they are not only metabolically dependent, but also depend on the position of the tissue. These motion‐induced changes in the CEST signal can be misinterpreted as metabolic activity [16].

Breath‐hold techniques offer a simple way to reduce motion but are not suitable for CEST MRI due to the long sequence duration and numerous required frequency offsets. Image‐based corrections, such as morphing kidneys between respiratory states, are hampered by CEST‐specific contrast changes and the high manual effort required. Jones et al. [17] proposed a retrospective method to reduce motion artifacts in lung CEST, using iterative short saturation pulses and excluding images outside the exhaled state, identified via k‐space phase differences in a liver‐dome ROI. Although this binning approach effectively reduced observed motion artifacts, the iterative saturation resulted in lower observed CEST effects compared to standard saturation, which could represent a potential limitation for renal CEST imaging where we expected small CEST effects [7]. Chen et al. [18] introduced a respiratory‐triggered approach for liver CEST by shortening the saturation period to fit within one breathing cycle, which improved homogeneity but yielded small CEST effects without validation using a static reference. A timed breathing strategy was previously proposed by Robson et al. [19] in the context of ASL imaging, in which participants synchronized their breathing with image acquisition to reduce motion artifacts. This approach has since been further evaluated in different renal CEST studies [20, 21, 22, 23, 24]. Wang et al. [23] reported a significant improvement in image quality for APT‐CEST using this timed breathing approach. Further, Wang et al. [24] demonstrated significant differences between healthy and malignant tissue in clear cell renal cell carcinoma (ccRCC) patients, highlighting the potential of renal CEST imaging for tumor characterization. The authors do not report any compartment‐specific differences in CEST effects, despite physiologically expected variations in metabolic processes. These differences may arise from a combination of factors, including Z‐spectrum sampling strategy, signal averaging, saturation parameters, and physiological influences such as hydration [25]. However, optimizing saturation parameters for renal CEST imaging is challenging, as validated simulation parameters are only rarely reported in the literature. Moreover, the timed breathing approach has not yet been systematically evaluated in the context of CEST imaging.

To address this issue, this feasibility study employed an in‐house‐developed electro‐pneumatic phantom simulating sequence‐synchronized kidney motion to validate the timed breathing strategy as a motion‐reduction approach for renal CEST MRI. The phantom setup enables direct comparison of motion‐affected data with a static ground truth, which is challenging to obtain in vivo, and allows systematic investigation of the feasibility and effects of applying the saturation module during the active phase of the respiratory cycle. In vivo Z‐spectra with 80 frequency offsets were acquired in healthy volunteers under both timed and free‐breathing conditions to assess exchange processes at 1.0, 2.0, and 3.5 ppm. To assess the clinical relevance of motion reduction and to explore the potential of renal CEST MRI in detecting pathology‐related metabolic differences, measurements were carried out in a patient with ccRCC under both breathing conditions.

## 2 Methods

### 2.1 Sequence Parameters

All measurements were performed on a 3 Tesla MRI system (Siemens Magnetom Prisma, Siemens Healthineers, Erlangen, Germany), using an 18‐channel body‐ and a 32‐channel spine‐coil (Body 18 SlideConnect and Spine 32 DirectConnect, Siemens Healthineers, Erlangen, Germany) in combination. To obtain *T*_2_‐weighted morphological reference images a half‐Fourier acquisition single‐shot turbo spin echo (HASTE) sequence was used. CEST measurements were conducted using an in‐house‐developed multi‐echo gradient echo sequence with a pulsed presaturation module. The CEST sequence was developed using the IDEA framework (versions VB17/VE11C, Siemens Healthineers, Erlangen, Germany) [26]. The parameters of the saturation module were selected based on prior experimental optimization, with the duty cycle maintained at a relatively low level to minimize potential SAR issues given the 100 ms saturation duration per pulse. The respective sequence parameters are listed in Table 1. The reference signal for normalizing the CEST signal was acquired at a frequency offset of 300 ppm. Two echoes were acquired to implement fat suppression in postprocessing using a two‐point Dixon method [7]. The saturation phase duration was 2.9 s, followed by an image acquisition of 0.7 s, resulting in a total sampling time of 3.6 s per image, which corresponds to a sampling rate of approximately 16.7 images per minute.

**TABLE 1:** Sequence parameters of the morphological images and the CEST images.

	Morphological images	CEST images
Sequence type	HASTE	GRE‐CEST
Echo time	99.0 ms	2.5 ms, 3.7 ms
Repetition time	1200.0 ms	5.4 ms
Flip angle	92.0°	15.0°
In‐plane FOV	(380.0 × 380.0) mm^2^	(380.0 × 380.0) mm^2^
Voxel size	(0.7 × 0.7 × 5.0) mm^3^	(3.0 × 3.0 × 5.0) mm^3^
Slices	20	1
Acquisition bandwidth	698 Hz/pxl	1220 Hz/pxl
Saturation (B_1_, *t*_pd_, *t*_ipd_, *n*_p_)	—	1.5 μT, 100 ms, 100 ms, 15
Frequency offset	—	±5.00 ppm
Number of frequency offsets	—	80
Averages	1	3
Acquisition time	24 s	14 min 36 s

*Note*: The CEST saturation parameters were abbreviated as follows: B_1_ (mean amplitude), *t*_pd_ (pulse duration), *t*_ipd_ (interpulse duration), *n*_p_ (number of pulses).

### 2.2 In Vitro Measurements

**FIGURE 1:**
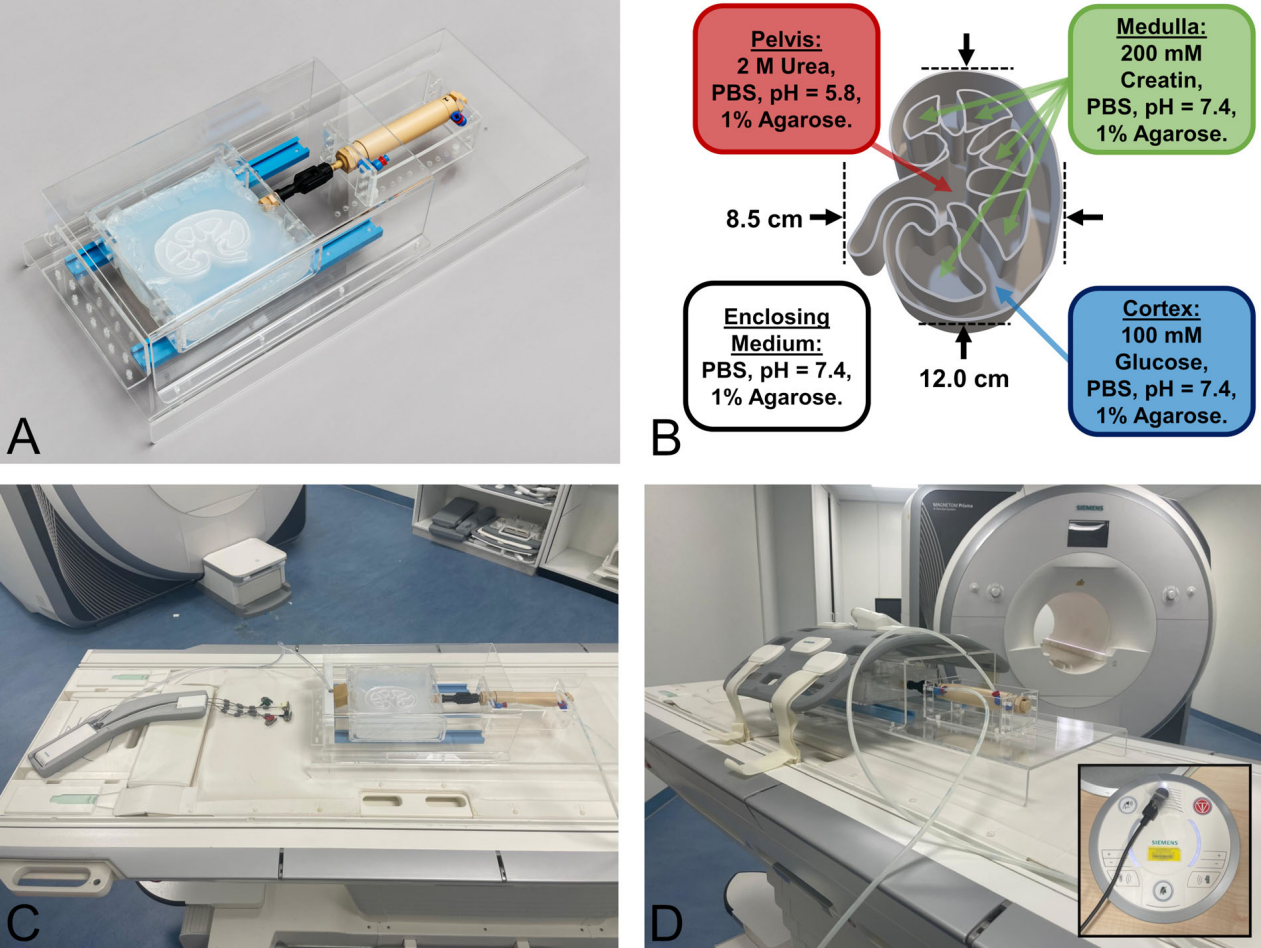
Image collage of the experimental setup for in vitro measurements. For the in vitro measurements, an electro‐pneumatic phantom with a 3D‐printed kidney CEST model was constructed (A). The kidney model (B) was filled with CEST metabolites and was moved during image acquisition. Motion was monitored using a respiratory cushion installed at the end of the motion rail, which was compressed by the foam during translation (C). Image acquisition was performed with the body coil mounted on the phantom and spine coil in combination (D). For timed motion, the control unit was triggered by the acquisition sound from the MRI console (subfigure in D).

An in‐house‐developed electro‐pneumatic phantom was used to investigate respiratory motion effects in CEST imaging (Figure 1A). The phantom experiments were inspired by Jones et al. [17] study design. The phantom allows periodic translational motion of a container holding the 3D‐printed kidney‐CEST model (.stl‐file can be provided on request). The phantom container, frame, and cover for coil placement were fabricated from 5 mm transparent PMMA plates and assembled with acrylic screws. Motion is guided by a custom 3D‐printed linear rail and driven by a double‐guided pneumatic cylinder. Compressed air is supplied to the pneumatic cylinder from outside the MR room via hoses and regulated by an electronically controlled valve. A Raspberry Pi running a Python script switches the valve periodically, enabling simulation of different respiratory cycles. The 3D‐printed kidney model (Figure 1B) was filled with 1% phosphate‐buffered agarose (ROTI Cell PBS pH 7.4 and ROTI Agarose, Carl ROTH GmbH & Co. KG, Karlsruhe, Germany). The choice of metabolites and their concentrations in the different compartments was guided by physiological considerations and WEX‐Studies of CEST metabolites in urine [6], given the limited availability of comprehensive quantitative data on compartment‐specific CEST‐active metabolite distributions in kidney tissue. The cortex was filled with 100 mM glucose (d(+)‐glucose monohydrate, Carl ROTH GmbH & Co. KG), reflecting the high perfusion of this compartment [5] and the known contribution of glucose to the CEST effect in blood [6, 27, 28]. The medulla contained 200 mM creatinine (anhydrous, Alfa Aesar, Haverhill, Massachusetts, USA), which was used instead of creatine due to the thermal instability of creatine [29] during agarose preparation. Both compounds exhibit CEST effects in the similar frequency range [6]. For the pelvis, the phosphate buffer was titrated to pH 5.8 using di‐potassium hydrogen phosphate and potassium dihydrogen phosphate, and the compartment was filled with 2 M urea (Carl ROTH GmbH & Co. KG). This choice was based on expected CEST effect of urea connected with urine [6, 30] and on preclinical evidence suggesting that the renal pelvis typically exhibits a lower pH [31]. Agarose was additionally added around the model to minimize susceptibility artifacts. Overall, the metabolite selection and concentrations were chosen to ensure a different detectable CEST contrast between compartments, while the primary purpose of the phantom was to demonstrate the feasibility of respiratory‐motion simulation rather than to exactly replicate kidney metabolism. The velocity of the periodic linear motion was set to approximately 10 cm/s via pneumatic throttles. To analyze the results of the CEST measurements as a function of the breathing rate, the motion amplitude of 10 mm was set, corresponding approximately to the renal displacement observed in previous studies [32, 33]. Motion frequency varied between [8.0:2.0:20.0] bpm [34] with an amplitude of 10 mm and with amplitudes of 5, 10, and 15 mm during sequence‐synchronized movement. The corresponding inhale and exhale dwell times were set to 1/3 and 2/3 of the breath period respectively [35]. To implement sequence‐synchronized movement of the phantom, a microphone was attached to the activated audio console (Figure 1D), and a motion cycle was triggered whenever the gradient‐induced sound level during acquisition exceeded a predefined intensity threshold. A breathing cushion was installed to monitor the motion (Figure 1C) and the body coil was installed above the kidney model (Figure 1D). B_0_ inhomogeneities were minimized by manual shimming. The shim setting was not changed during all in vitro experiments.

### 2.3 In Vivo Measurements

In the vivo measurements, 10 healthy volunteers (six females, four males, mean age 25.2 ± 1.9 years) and one patient (male, 47 years) with a (3.0 × 2.2 × 2.2) cm^3^ histologically confirmed ccRCC were examined with the same sequence protocol as in in vitro measurements. Since the right and left kidneys showed comparable motion in the coronal plane across an entire cohort (study by Song et al. [32] with 10 volunteers; right: 8.9 ± 3.7 mm; left: 8.48 ± 3.04 mm), the right kidney was selected to enable precise placement of the image plane through the respective kidney and to ensure comparability between healthy subjects and the patient whose right kidney was affected by ccRCC. Written informed consent was obtained from all participants and the study was approved by the local ethics committee (Ethics Committee, Medical Faculty of the Heinrich‐Heine‐University Düsseldorf; healthy controls: study number 2022‐1913_1, patient: study number 2022‐1913). All subjects were positioned head‐first in the supine position. A breathing cushion monitored abdominal motion to determine the breathing rate. A manual shim was performed to minimize B_0_ inhomogeneities. All subjects were examined twice with the CEST sequence. In the first CEST measurement, the volunteers were asked to breathe freely. For the second measurement, the subjects were instructed to time their breathing in accordance with the image acquisition of the CEST sequence. The image acquisition was audible to the volunteers as a loud buzzing noise, while the saturation phase was audible as clicking noises. During the saturation phase, the volunteers were allowed to breathe, while maintaining their exhaled state during image acquisition, to minimize the influence of breathing on the CEST image series.

### 2.4 Postprocessing

**FIGURE 2:**
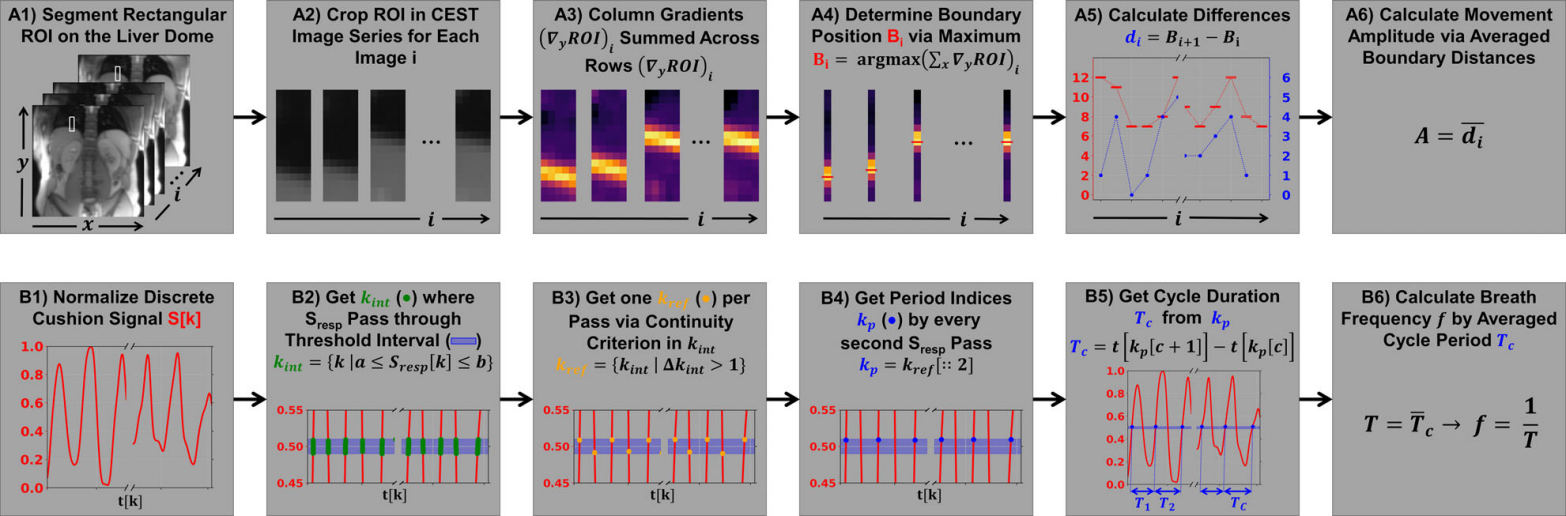
Methodology for analysis of breathing motion and breathing frequency. A rectangular ROI at the liver dome (A1) was cropped filtered with a median filter (A2) and gradient filter (A3), and the lung–liver boundary was identified from summed row intensities (A4). Frame‐to‐frame displacements (A5) were used to calculate the mean absolute respiratory motion (A6). The normalized respiratory signal (B1) from the breathing cushion was thresholded (blue area, B2), and crossings were identified (green dots). From these, reference indices were selected (orange dot, B3) using a continuity condition. Each breathing period was calculated by every second reference index (blue bullets, B4), from which the average respiratory frequency was calculated (B5, B6).

Data postprocessing and results visualization was conducted with in‐house developed Python 3.10 scripts. Respiratory motion was quantified in vivo by liver‐lung boundary [17, 32] or phantom edge displacement within a ROI placed at the liver dome or phantom edge. The lung liver boundary and the phantom edge were identified using an edge detection algorithm (Figure 2A). The edge detection algorithm cropped the segmented ROI at the lung‐liver interface and applied a 3 × 3 median filter (using the SciPy [36] built‐in function ndimage.median_filter()) to suppress small structures, such as bronchi or vessels, that could be erroneously detected as edges. Column‐wise derivatives were then calculated using second‐order accurate central differences (using the NumPy [37] built‐in function numpy. gradient()). The row‐wise sums were calculated, and the maximum sum was taken as the lung–liver edge position. The breathing rate was determined in postprocessing by analyzing the periodicity of the breathing cushion signal (Figure 2B). The open‐source software fmri‐physio‐log [38] was used to import and read out the respiratory cushion data.

To register the HASTE images on a CEST image in the exhaled state and segment ROIs for cortex, medulla, pelvis, liver dome and ccRCC tumor, the open source software ITK snap [39] was used. The segmentation was performed by A.L. (radiologist, 10 years of experience).

For fat suppression water‐only‐images were calculated using the Dixon method and retained for CEST analysis [7]. A Gaussian filter (*σ* = 0.75) was applied to the water‐only‐images to reduce the influence of image noise [40]. B_0_‐inhomogeneity was corrected by interpolating the Z‐spectrum in 0.01 ppm steps using a local tangent (0.5 ppm interval) and realigning to the spectral minimum, following the Z‐spectrum‐based correction approach recommended by Chen et al. [18]. The magnetization transfer ratio asymmetry (MTR_asym_) was used to quantify the CEST effect in the Z‐Spectra. The MTR_asym_ calculation approach is based on the method described by Jones et al. [17] and uses the normalized signal intensity S at the frequency offset Δω, which is determined by a linear fit of the data points in the Z‐spectra within the interval [Δω±0.5]. Outgoing from the fitted slope m and intersection b, the signal intensity S at Δω was determined by S(Δω)=m·Δω+b. Afterwards, MTR_asym_ was calculated by

$${\mathrm{MTR}}_{\text{asym}}\left(\Delta \omega \right)=\frac{S\left(-\Delta \omega \right)-S\left(\Delta \omega \right)}{S_{0}}$$

pixel wise at 1.0, 2.0, and 3.5 ppm, as the exchange of glucose, creatine, creatinine, urea, and APT protons is expected in these frequency offsets [6, 7].

### 2.5 Statistics

#### 2.5.1 In Vitro Statistics

The mean absolute error (MAE) was used to quantify the deviation between data with motion and static ground truth. To calculate the MAE, the ROI‐averaged $\bar{\mathrm{MTR}}$_asym_(Δω) in the results $X_{\left(A,f\right)}$ (with motion amplitude A and frequency f) and the results in the static ground truth results $Y_{\left(\mathrm{GT}\right)}$ were considered.

$$\begin{array}{cc} & X_{\left(A,f\right)}=\{{\bar{\mathrm{MTR}}}_{\text{asym}}\left(1.0\;\mathrm{ppm};\text{Cortex}\right),{\bar{\mathrm{MTR}}}_{\text{asym}}\left(1.0\;\mathrm{ppm};\text{Medulla}\right),\\ & \;{\bar{\mathrm{MTR}}}_{\text{asym}}\left(1.0\;\mathrm{ppm};\text{Pelvis}\right),\ldots \\ & \;\ldots ,{\left.\;{\bar{\mathrm{MTR}}}_{\text{asym}}\left(3.5\;\mathrm{ppm};\text{Medulla}\right),{\bar{\mathrm{MTR}}}_{\text{asym}}\left(3.5\;\mathrm{ppm};\text{Pelvis}\right)\right\}}_{\left(A,f\right)}\\ & \;=\left\{{\bar{\mathrm{MTR}}}_{\text{asym}}\right.\left(∆\omega ;r\;\right)|∆\omega \in \{1.0\;\mathrm{ppm},2.0\;\mathrm{ppm},\;3.5\;\mathrm{ppm}\}\;,\\ & \;r\in \{\text{Cortex}\;\;,\text{Medulla},{\;\;\text{Pelvis}\}\}}_{\left(A,f\right)}\\ & Y_{\left(\mathrm{GT}\right)}=\left\{{\bar{\mathrm{MTR}}}_{\text{asym}}\right.\left(∆\omega ;r\;\right)|∆\omega \in \{1.0\;\mathrm{ppm},2.0\;\mathrm{ppm},\;3.5\;\mathrm{ppm}\}\;,\\ & \;r\in \{\text{Cortex}\;\;,\text{Medulla},{\;\;\text{Pelvis}\}\}}_{\left(\mathrm{GT}\right)} \end{array}$$

Accordingly, the MAE for each experiment (A,f) was calculated by

$$\mathrm{MAE}\left(A,f\right)=\mathrm{MAE}\left(X_{\left(A,f\right)},Y_{\left(\mathrm{GT}\right)}\right)=\frac{1}{9}\;\sum_{i=1}^{9}\left|X_{{\left(A,f\right)}_{i}}-Y_{{\left(\mathrm{GT}\right)}_{i}}\right|.$$

Fisher transformation and standard deviation were used to calculate 95% confidence intervals.

#### 2.5.2 In Vivo Statistics

The normal distribution of the averaged MTR_asym_ values in the healthy subjects was tested for the cortex, medulla and pelvis using a Shapiro–Wilk Test (*α* = 0.01). The ROI averaged MTR_asym_ values of the ROIs with timed and free breathing were then compared using a paired *t*‐test with Bonferroni correction. The ROI averaged MTR_asym_ values of the patients were compared to the healthy subjects using a one‐sample *t*‐test with Bonferroni correction. Significance levels were defined by (****) for *p* ≤ 0.0001, (***) for 0.0001 < *p* ≤ 0.001, (**) for 0.001 < *p* ≤ 0.01, (*) for 0.01 < *p* ≤ 0.05, and not significant (ns) for 0.05 < *p*. All statistical analyses were performed using the Python library SciPy [36, 41].

## 3 Results

### 3.1 In Vitro Results

**FIGURE 3:**
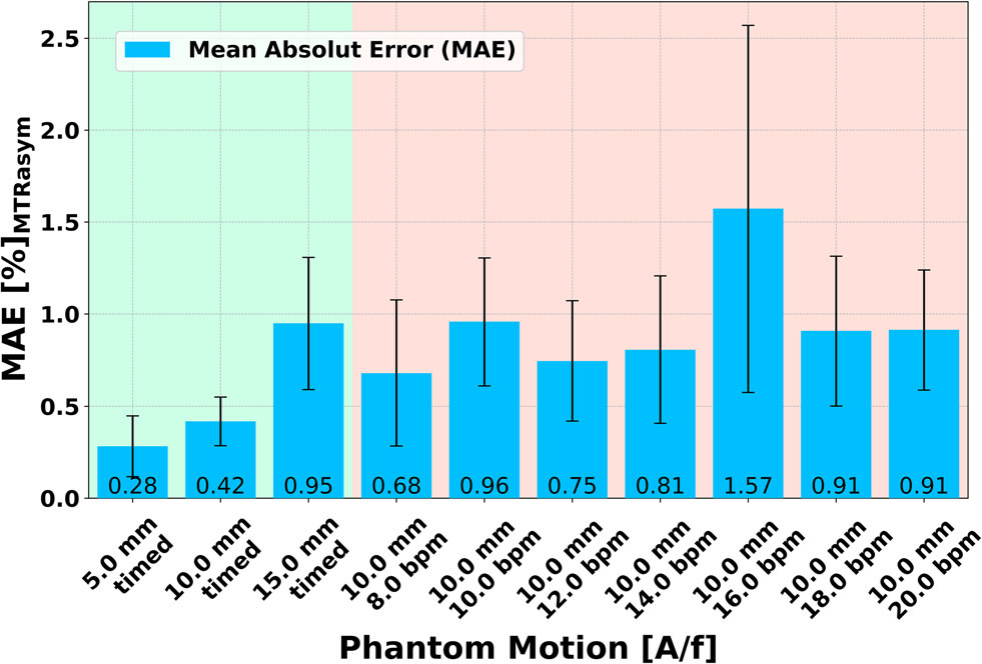
Bar chart to validate the timed motion. To validate timed breathing with a static ground truth, the MTR_asym_ values for ROI and MTR_asym_ offset were averaged respectively in vitro and the Mean Absolut Error (MAE) to the ground truth (GT) were calculated. The results of the timed motion are highlighted in green and those with different motion periods are highlighted in red. Fisher transformation and standard deviation was used to calculate 95% confidence intervals.

The experiments with the timed motion up to an amplitude of 10 mm show the lowest MAE compared to the experiments with a fixed motion period (Figure 3). The measurements with 16.0 bpm show maximum MAE, while 8.0 bpm shows the minimum MAE for periodic motion.

**FIGURE 4:**
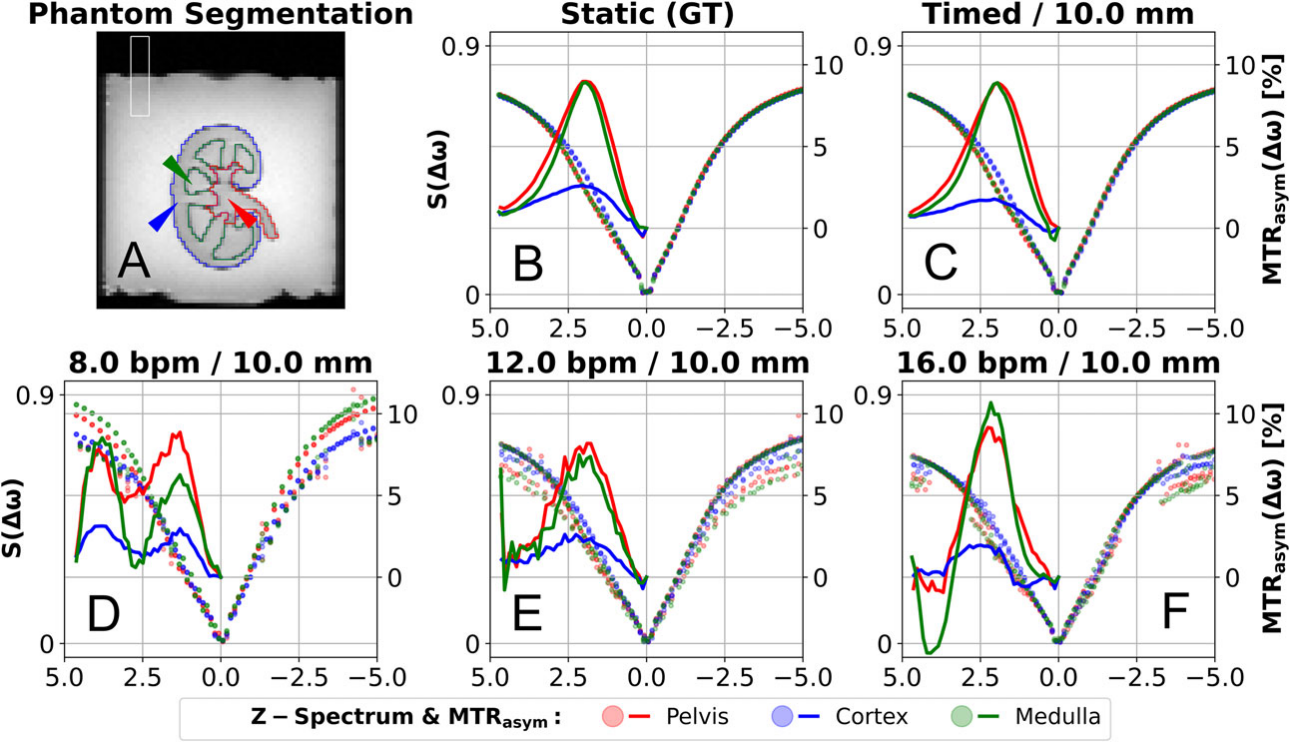
Segmentation and an exemplary Z‐spectra in vitro. Morphological image (top left) was registered to CEST images in exhaled position, and segmentations were performed for cortex (blue), medulla (green), and pelvis (red) in the kidney CEST model. Furthermore, a ROI was segmented at the phantom edge (white) to monitor the motions in the CEST images. The Z‐spectra of the exemplary selected pixels (wedges in HASTE images) show a correlation between static measurement (top center) and timed motion (top right). For periodic motions of 8.0, 12.0, and 16.0 bpm (corresponding order below), discontinuous Z‐spectra and pseudo‐CEST effects are discernible in the MTR_asym_ curves.

The in vitro Z‐spectrum and MTR_asym_ curves between static (Figure 4B) and timed measurement (Figure 4C) show a similar profile. In the Z‐spectrum of the cortex with timed motion, a slight decrease in the observed CEST effect is visible compared to the static measurement. The exemplary periodic motions of 8.0, 12.0, and 16.0 bpm (Figure 4D–F) are characterized by noisier Z‐spectra, and the resulting MTR_asym_ curves exhibit distinct pseudo‐CEST effects. Due to free movement, the position of the normalized image differs at 300 ppm, resulting in a different normalization of the Z‐spectrum at 8 bpm (Figure 4D).

**FIGURE 5:**
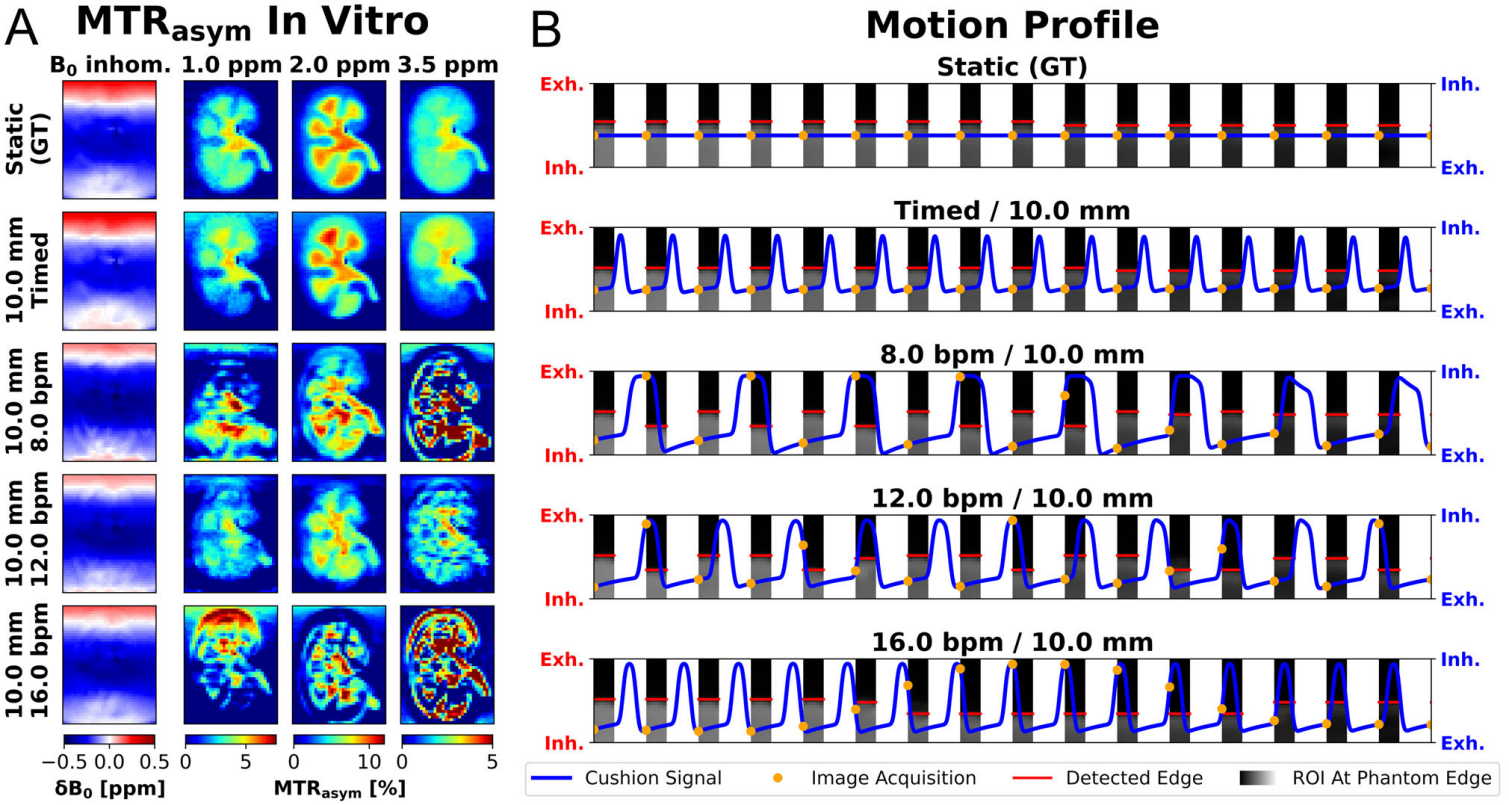
In vitro MTR_asym_ maps and motion profile section. In (A) the determined B_0_ inhomogeneities and corresponding MTR_asym_ maps are shown for the static measurement, timed motion and periodic motion frequency of 8.0, 12.0, and 16.0 bpm and 10.0 mm motion amplitude (corresponding order from top to bottom). Based on the recorded cushion signal (blue solid line) and acquisition time tag (orange dot) in (B), the CEST images were assigned to the motion states. For each CEST image, a ROI was analyzed at the phantom edge (image section in gray scales) and the phantom edge (red horizontal line) was detected using an edge detection algorithm.

In the MTR_asym_ maps in Figure 5A, the individual kidney model compartments are visible in the static measurement and with timed motion. In timed motion, a MTR_asym_ gradient is visible along the direction of motion. At motion frequencies of 8.0 and 16.0 bpm, cluster‐like MTR_asym_ artifacts appear which are visually related to the structure of the renal CEST model. At 12.0 bpm, the MTR_asym_ maps show CEST effects in the shape of the kidney model without depicting the compartments and increased MTR_asym_ values in the pelvis.

The phantom edge could be correctly identified and the images assigned to the correct positions in the respiratory profile (Figure 5B). At 8.0 and 16.0 bpm, the image acquisition shows aliasing effects in which successive images are acquired in groups in the same breathing state. The positions in which the phantom is acquired change more frequently at 12.0 bpm.

### 3.2 In Vivo Results

The averaged respiratory motion in the CEST images was significantly reduced with timed breathing (*p* < 0.0001), decreasing from (10.0 ± 4.3) mm to (2.0 ± 1.2) mm. The average free breathing rate was (12.1 ± 2.8) bpm and was adapted to (16.4 ± 0.4) bpm with timed breathing.

**FIGURE 6:**
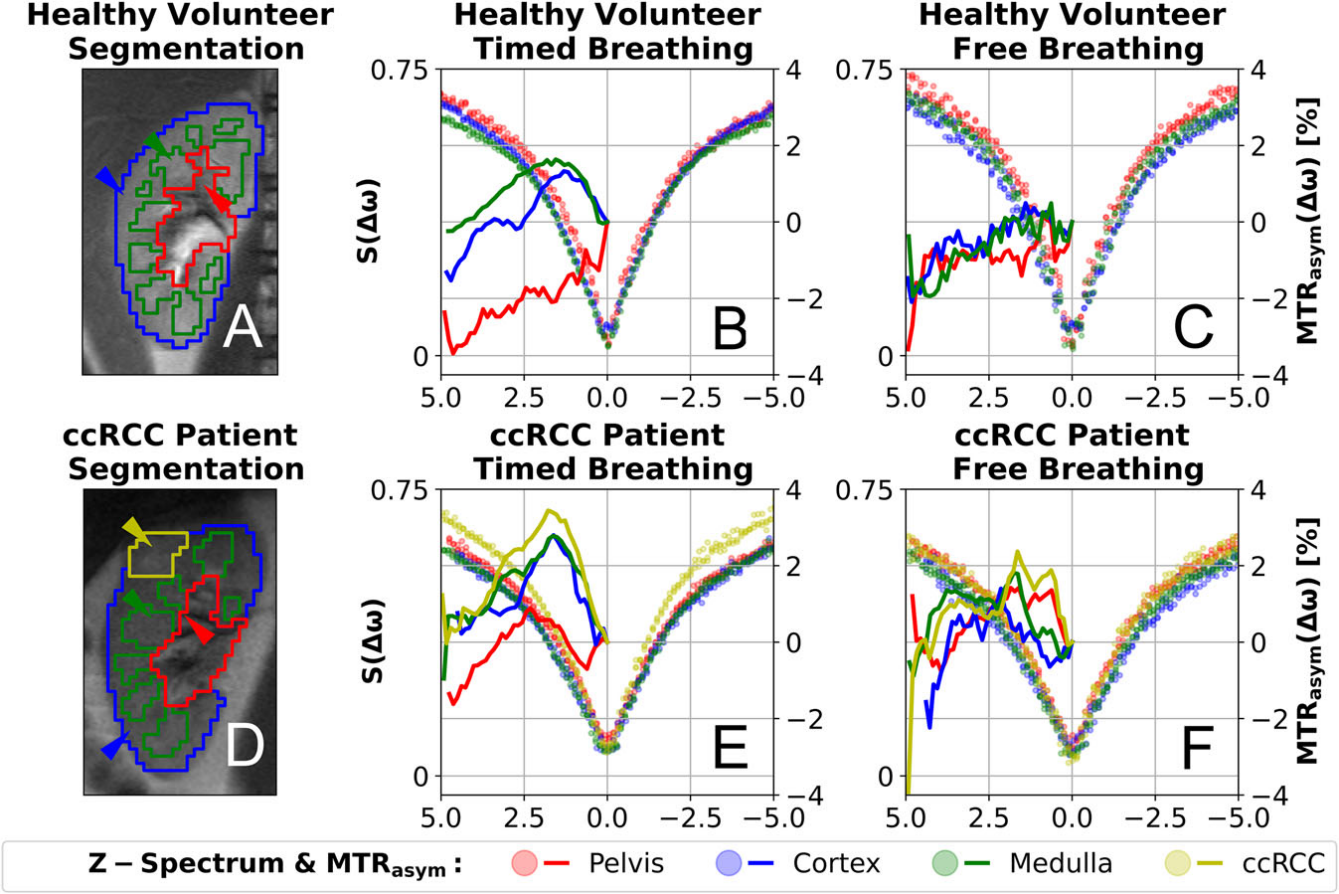
Segmentation and an exemplary Z‐spectrum in vivo. Morphological images were registered to the exhaled CEST images and segmentations were performed for cortex (blue), medulla (green), pelvis (red) and ccRCC (yellow) for a healthy subject (A) and a patient with ccRCC (D). The Z‐spectra of the exemplary selected pixels (wedges in A and D) with timed breathing are shown, for the healthy volunteer (B) and the patient (E) respectively. The same pixels are shown with free breathing for the healthy volunteer (C) and the patient (F).

With free breathing, the Z‐spectra (Figure 6C,D) show a pronounced scattering of the data points, and the resulting MTR_asym_ curves are noisy. Using timed breathing significantly reduces this scatter, resulting in smoother Z‐spectra and MTR_asym_ profiles (Figure 6B,D). This improvement is particularly noticeable in the cortex and medulla, while it is less pronounced in the pelvis.

**FIGURE 7:**
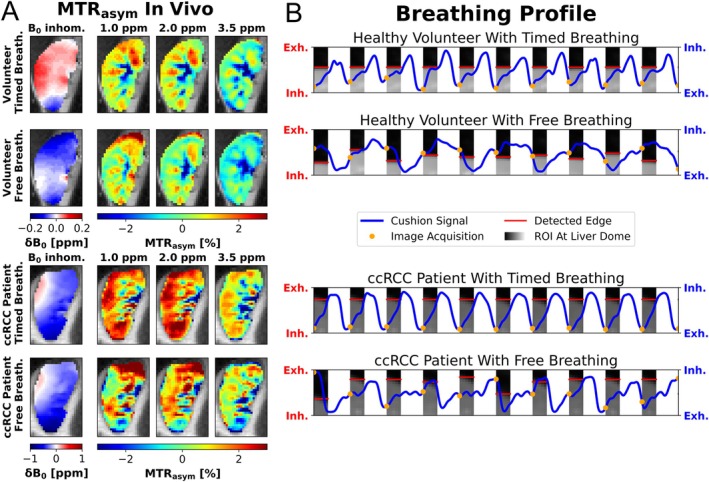
In vivo MTR_asym_ maps and breathing profile section. In (A) the B_0_ inhomogeneity and corresponding MTR_asym_ maps are shown for timed breathing and free breathing frequency in a healthy volunteer (top) and a ccRCC patient (bottom) with outlined ROIs (black). Based on the recorded respiratory cushion signal (blue solid line) and acquisition time tag (orange dot) in (B), the CEST images were assigned to respiratory states. For each CEST image, a ROI was analyzed at the liver dome (image section in gray scales) and the lung‐liver boundary was detected using an edge‐detection algorithm (red horizontal line).

The B_0_ inhomogeneity profile is not discernible in the MTR_asym_ maps with timed breathing (Figure 7A). Structures of the medullary pyramids and the renal pelvis are visible in the MTR_asym_ maps of the healthy volunteer obtained with timed breathing. These structures appear blurred under free breathing conditions. Increased MTR_asym_ values at 2.0 ppm in the upper cortex suggest motion‐related artifacts. Compared to the MTR_asym_ maps of the healthy volunteer with timed breathing, the structural details in the patient appear less distinct.

The breathing profiles (Figure 7B) confirm that the image acquisition was correctly perceived and that motion in the CEST image series was effectively reduced by timed breathing. With free breathing, images were acquired in the exhaled and inhaled states, and the motion of the lung–liver boundary is evident.

**FIGURE 8:**
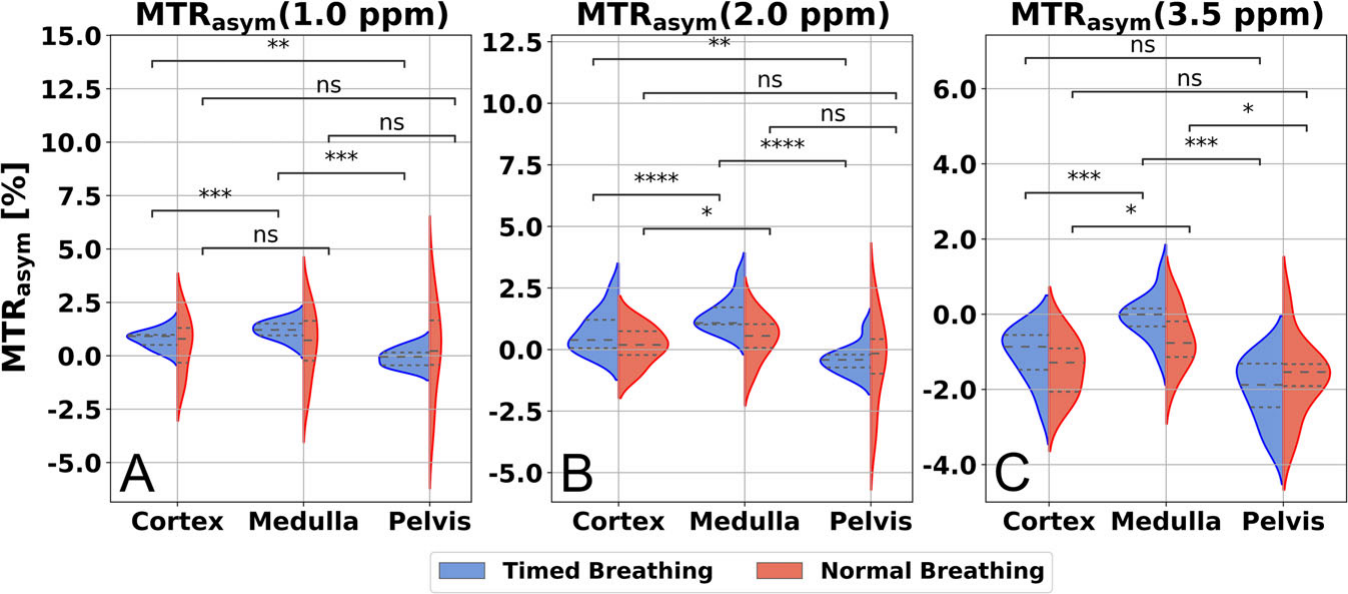
Violin plot of the in vivo MTR_asym_ results. The ROI averaged MTR_asym_ values by healthy volunteers at 1.0 (A), 2.0 (B), and 3.5 ppm (C) with timed (blue) and free (red) breathing were statistically compared using a paired *t*‐test. Significance levels were defined by (****) for *p* ≤ 0.0001, (***) for 0.0001 < *p* ≤ 0.001, (**) for 0.001 < *p* ≤ 0.01, (*) for 0.01 < *p* ≤ 0.05, and (ns) for 0.05 < *p*.

The Shapiro–Wilk Test confirmed a normal distribution of ROI averaged MTR_asym_ values in the healthy volunteers (Figure 8). With timed breathing, there is a significant difference (*p* ≤ 0.01) in the MTR_asym_ values at 1.0 ppm between the medulla, cortex, and pelvis, which are not significant with free breathing (Figure 8A). At 2.0 ppm there is a significant difference in the MTR_asym_ values between the cortex, medulla and pelvis with timed breathing (*p* ≤ 0.01) (Figure 8B). There is no significant difference in MTR_asym_ values at 3.5 ppm between the cortex and pelvis (Figure 8C). The MTR_asym_ difference at 3.5 ppm between the cortex and medulla, as well as medulla and pelvis, is significant both with timed breathing (*p* ≤ 0.001) and free breathing (*p* ≤ 0.05).

At 1.0 ppm MTR_asym_ values with timed breathing of the ccRCC are significantly increased compared to the mean values of the healthy subjects in the cortex (*p* ≤ 0.0001), medulla (*p* ≤ 0.001), and pelvis (*p* ≤ 0.0001). These significant differences are not obtained with free breathing. A significantly increased MTR_asym_ value at 2.0 ppm of ccRCC compared to the healthy subjects was found with timed breathing in the cortex (*p* ≤ 0.001), medulla (*p* ≤ 0.01), and pelvis (*p* ≤ 0.0001). The MTR_asym_ value at 3.5 ppm of the ccRCC was significantly increased compared to the healthy subjects in the cortex (*p* ≤ 0.0001), medulla (*p* ≤ 0.01), and pelvis (*p* ≤ 0.0001). With timed breathing, significantly increased MTR_asym_ values in the kidney compartments were obtained between healthy subjects and patients, except for the medulla at 2.0 ppm (Table 2).

**TABLE 2:** MTR_asym_ results in the in vivo study.

MTR_asym_ (%)		Healthy collective (*n* = 10)		ccRCC patient (*n* = 1)	
Offset (ppm)	ROI	Free	Timed	Free	Timed
1.0	Cortex	0.53 ± 1.30	0.78 ± 0.41	1.17 ± 2.63^(ns)^	1.38 ± 1.25^(**)^
	Medulla	0.57 ± 1.56	1.20 ± 0.42	0.87 ± 1.62^(ns)^	1.71 ± 0.86^(*)^
	Pelvis	0.32 ± 2.19	−0.09 ± 0.42	0.60 ± 1.94^(ns)^	0.50 ± 1.75^(**)^
	ccRCC	—	—	1.39 ± 1.43	2.17 ± 0.63
2.0	Cortex	0.15 ± 0.76	0.70 ± 0.83	0.99 ± 1.75^(*)^	1.54 ± 1.26^(*)^
	Medulla	0.46 ± 0.88	1.43 ± 0.70	1.09 ± 1.25^(ns)^	2.06 ± 0.81^(ns)^
	Pelvis	−0.41 ± 1.75	−0.39 ± 0.58	0.82 ± 1.83^(ns)^	0.60 ± 1.87^(**)^
	ccRCC	—	—	1.11 ± 0.73	2.49 ± 0.70
3.5	Cortex	−1.43 ± 0.77	−1.09 ± 0.71	−0.25 ± 1.64^(**)^	0.23 ± 1.27^(***)^
	Medulla	−0.67 ± 0.77	−0.07 ± 0.58	0.35 ± 1.02^(**)^	1.07 ± 0.57^(***)^
	Pelvis	−1.70 ± 1.00	−2.02 ± 0.84	−0.63 ± 1.99^(*)^	−1.23 ± 2.09^(*)^
	ccRCC	—	—	0.48 ± 0.99	1.04 ± 0.42

*Note*: Significant differences between the patient and the healthy collective were determined using a one‐sample *t* test for both timed and free breathing. In the healthy collective, the error was calculated using the standard error of the mean. The error in the patient's results was determined using the standard deviation of the MTR_asym_ values measured in the respective ROI. Significance levels were defined by (****) for *p* ≤ 0.0001, (***) for 0.0001 < *p* ≤ 0.001, (**) for 0.001 < *p* ≤ 0.01, (*) for 0.01 < *p* ≤ 0.05 and (ns) for 0.05 < *p*.

## 4 Discussion

The methodology for timed breathing was successfully evaluated in an electro‐pneumatic phantom. The in vivo MTR_asym_ maps with timed breathing revealed contrast differences that could be assigned to distinct renal compartments, demonstrating the potential of CEST MRI to visualize different renal metabolic processes. The patient pilot study indicates that renal CEST imaging may provide metabolic information related to pathological changes.

Our phantom study highlights the challenges associated with developing and applying existing respiratory correction methods in renal CEST imaging. The retrospective exclusion of images acquired outside a defined respiratory state, as proposed by Jones et al. [17], can lead to signal gaps in the Z‐spectrum, resulting in pseudo‐CEST effects. This is evident when comparing data acquired at 8 and 16 bpm to a static reference. The gaps in the Z‐spectrum can be explained by the fact that successive images often occur in similar respiratory states, which gradually change over time when there are only minimal deviations between the motion frequency and the sampling frequency (see Animation, Supplemental Digital Content 2, which interactively simulates image acquisition along a sinusoidal breathing curve). This effect can be mitigated when the sampling frequency is not close to the motion or respiratory frequency, as suggested by our phantom results at 12 bpm. In this case, the overall MTR_asym_ profile is largely preserved, albeit with increased instability. This instability could likely be reduced by increasing the sampling density, as implemented in the iterative saturation approach by Jones et al. [17]. However, iterative saturation has been reported to reduce the observed CEST effect [17], raising concerns as to whether the contrast differences detected in the present study would remain observable under iterative saturation.

Timed breathing reduces motion artifacts without compromising the CEST effect and can be implemented vendor‐independently. Nevertheless, residual motion remains, related to other physiological processes such as intestinal and ureteral peristalsis or arterial pulsation [7], which cannot be compensated by timed breathing. Fluid‐induced motion is suggested in the renal pelvis, where the Z‐spectrum and MTR_asym_ curves show less improvement with timed breathing, which may be attributed to urinary flow.

Previous studies [18, 21, 23] on abdominal CEST imaging have primarily focused on conducting saturation and acquisition during the exhaled phase of the breathing cycle. While this approach minimizes motion, it limits the saturation duration. In our study, saturation was applied during active breathing. No motion‐related artifacts were observed in vivo, although phantom experiments showed deviations between timed motion and the static ground truth. We hypothesize that differences in the static magnetic field during saturation between the moving and static states may lead to these discrepancies, as the observed deviations in MTR_asym_ resemble the field inhomogeneity profile. However, since such patterns were not evident in the in vivo data, the practical relevance of this effect observed in vitro for in vivo CEST imaging appears limited. Nevertheless, we recommend that this potential limitation be taken into account in future developments of respiratory‐triggered CEST sequences, for instance through improved shim homogeneity or developing suitable correction methods.

Our patient results align with Wang et al. [24], who reported increased APT‐CEST in ccRCC and also reinforces their used timed breathing approach for motion reduction, as free‐breathing acquisitions may lead to misinterpretation of metabolic information [16]. Their used timed breathing approach differs slightly from our used approach; Wang et al. [24] used explicit breathing commands, whereas in our approach participants synchronized breathing directly to the acoustic cues of the sequence (saturation “clicking” vs. acquisition “buzzing”). The timed breathing protocol by Wang et al. [24] used a 10 s cycle (≈6 bpm), near the physiological lower limit [34] and potentially demanding for the subjects (especially for dyspneic patients), thus restricting Z‐spectrum sampling. We therefore suggest adapting the timed breathing protocol to natural respiration rates to improve comfort and thus spectral resolution in Z‐spectra.

Timed Breathing effectively pursues the same approach as navigator‐based techniques (i.e., image acquisition in the exhaled state) which has been shown to be effective for motion reduction in other renal MRI applications [32, 42, 43, 44]. Nevertheless, our observations indicate that some residual motion may persist in timed‐breathing image series, likely related to peristaltic activity. Therefore, combining timed breathing with postprocessing motion correction could potentially improve the mitigation of motion beyond the translational motion of the kidney caused by respiration. In this context, Wang et al. [24] have emphasized an structural mutual information (SMI)‐based registration approach [45], which could be further evaluated in future CEST studies.

To the best of our knowledge, no study has examined endogenous CEST contrast between native kidney compartments. Thus, the observed differences can only be hypothesized based on physiological findings. A physiological suggestion for the increased CEST effect of (0.78% ± 0.41%) in the healthy cortex at 1.0 ppm could be that the renal cortex receives about 90% of the renal blood flow [5] containing about 3.9–5.6 mM glucose [28], which shows a CEST effect around 1.2 ppm [6]. The observed MTR_asym_ values of (1.43% ± 0.70%) in the medulla at 2.0 ppm may reflect creatinine enrichment through the glomerular filtration [5, 6]. In addition, creatine, alanine, glutamine, and allantoin are expected metabolites in the kidney and could contribute to the increased MTR_asym_ value at 2.0 ppm [6]. Due to the MTR_asym_ calculations and a potential nuclear overhauser effect (NOE) at −3.5 ppm [46], the MTR_asym_ maps at 3.5 ppm may reflect a superposition of APT‐CEST and NOE effects. As the MTR_asym_ values at 3.5 ppm are negative in some kidney compartments, it can be assumed that the NOE effect is more dominant than the physical proton‐exchange‐based CEST effect in these areas. Since both mechanisms are pH‐dependent [8, 9, 47], the observed contrast differences may reflect varying pH values across renal compartments, consistent with findings from preclinical [31] and initial human Iopamidol‐CEST studies [48] showing pH differences between cortex and medulla.

In addition to increased MTR_asym_ values in the patient compared to the healthy cohort, renal compartments appear less distinctly and with a global rather than lesion‐specific increase of MTR_asym_. A potential explanation for these findings may be paraneoplastic metabolic alterations associated with ccRCC, such as enhanced lactate metabolism [49], which can also be assessed using CEST imaging [50], or pH changes mediated by the expression of proteins such as carbonic anhydrase 9 [14]. Furthermore, altered diffusion properties of the kidney in the context of ccRCC [15, 51] may facilitate a global manifestation of these metabolic changes, potentially accounting for the observed results.

Our study has limitations that are worth noting. First, when comparing the MTR_asym_ values, it should be noted that the CEST effect may not be influenced by metabolite concentration and exchange only, but also by the *T*_1_ relaxation time [52]. In the kidney, longer *T*_1_ times were measured in the medulla than in the cortex [53], which could provide different MTR_asym_ values. Although a lower pH value in the medulla is expected [31], this should, in turn, reduce the CEST effect of for example, creatinine [6]. These contradictory influences require further study, as *T*_1_ correction methods have not yet demonstrated any significant differences [18] in abdominal CEST and its relevance in renal imaging remains unclear. Secondly, our phantom only partially reflects in vivo conditions, as real kidney motion involves complex deformation and differences in shimming limit comparability with in vivo experiments. The implementation of a more homogeneous shim is challenging due to the material‐related, geometric construction and associated susceptibility properties of the phantom. Third, our in vivo study included a small, mostly young cohort and one patient, limiting broader applicability, as timed breathing requires substantial compliance and can be challenging for renal patients with shortness of breath.

The clinical use of timed breathing with the current sequence is limited by its long acquisition time (14 min 36 s). Nevertheless, our results indicate that extending saturation into the active respiratory cycle is feasible without visible artifacts, which may be relevant for the development of future CEST sequences. To the best of our knowledge, this study is the first to demonstrate significant CEST contrast between individual renal compartments, highlighting the potential of metabolically sensitive renal imaging to differentiate healthy from pathological tissue. In the future, protocol optimization using CEST simulations for kidney‐relevant metabolites [6], the adaptation of denoising methods [40, 54] to improve signal efficiency, alternative sampling strategies [55, 56] and the implementation of fast 3D CEST sequences, such as snapshot‐CEST [57, 58] may enable faster and clinically feasible renal CEST imaging.

## 5 Conclusion

The phantom enabled successful validation of the timed breathing approach for motion reduction in renal CEST imaging and revealed potential methodological limitations. This study demonstrates the feasibility of renal CEST imaging by minimizing respiratory motion. Preliminary measurements in a ccRCC patient emphasize the importance of motion reduction in CEST data for a potential clinical application of renal CEST imaging.

## Funding

This work was supported by the Jürgen Manchot Stiftung.

## Supporting information


**Supplemental Digital Content 1.** phantom.mp4. The supplementary video shows exemplary the motion of the electro‐pneumatic phantom with the installed breathing cushion and kidney CEST model.

**Supplemental Digital Content 2.** sampling_animation.html. The HTML file is an interactive animation illustrating image acquisition during a CEST scan along a regular sinusoidal breathing curve.

## Data Availability

The data that support the findings of this study are available from the corresponding author upon reasonable request.

## References

[1] S. Copur , F. Yavuz , A. A. Sag , K. R. Tuttle , and M. Kanbay , “Future of Kidney Imaging: Functional Magnetic Resonance Imaging and Kidney Disease Progression,” European Journal of Clinical Investigation 52, no. 5 (2022): 2–4, 10.1111/eci.13765. 35267195

[2] K. Ward , A. Aletras , and R. Balaban , “A New Class of Contrast Agents for MRI Based on Proton Chemical Exchange Dependent Saturation Transfer (CEST),” Journal of Magnetic Resonance 143, no. 1 (2000): 79–87, 10.1006/jmre.1999.1956. 10698648

[3] L. Liu , S. Guo , Z. Xing , et al., “Chemical Exchange Saturation Transfer Magnetic Resonance Imaging of the Kidney: Applications and Challenges,” Abdominal Radiology 50 (2025): 5934–5947, 10.1007/s00261-025-04980-2. 40448845 PMC12602669

[4] B. Wu , G. Warnock , M. Zaiss , et al., “An Overview of CEST MRI for Non‐MR Physicists,” EJNMMI Physics 3, no. 1 (2016): 19, 10.1186/s40658-016-0155-2. 27562024 PMC4999387

[5] J. S. Meltzer , “Renal Physiology,” in Pharmacology and Physiology for Anesthesia, ed. H.C. Hemmings , and T.D. Egan (Elsevier, 2019), 782–794, 10.1016/B978-0-323-48110-6.00040-5.

[6] J. Stabinska , P. Neudecker , A. Ljimani , H. Wittsack , R. S. Lanzman , and A. Müller‐Lutz , “Proton Exchange in Aqueous Urea Solutions Measured by Water‐Exchange (WEX) NMR Spectroscopy and Chemical Exchange Saturation Transfer (CEST) Imaging In Vitro,” Magnetic Resonance in Medicine 82, no. 3 (2019): 935–947, 10.1002/mrm.27778. 31004385

[7] J. Stabinska , A. Müller‐Lutz , H. J. Wittsack , et al., “Two Point Dixon‐Based Chemical Exchange Saturation Transfer (CEST) MRI in Renal Transplant Patients on 3 T,” Magnetic Resonance Imaging 90 (2022): 61–69, 10.1016/j.mri.2022.04.004. 35476934

[8] K. J. Ray , M. A. Simard , J. R. Larkin , et al., “Tumor pH and Protein Concentration Contribute to the Signal of Amide Proton Transfer Magnetic Resonance Imaging,” Cancer Research 79, no. 7 (2019): 1343–1352, 10.1158/0008-5472.CAN-18-2168. 30679178 PMC6462213

[9] J. Schüre , M. Shrestha , S. Breuer , et al., “The pH Sensitivity of APT‐CEST Using Phosphorus Spectroscopy as a Reference Method,” NMR in Biomedicine 32, no. 11 (2019): e4125, 10.1002/nbm.4125. 31322308

[10] K. Pavuluri , I. Manoli , A. Pass , et al., “Noninvasive Monitoring of Chronic Kidney Disease Using pH and Perfusion Imaging,” Science Advances 5, no. 8 (2019): eaaw8357, 10.1126/sciadv.aaw8357. 31453331 PMC6693904

[11] D. L. Longo , J. C. Cutrin , F. Michelotti , P. Irrera , and S. Aime , “Noninvasive Evaluation of Renal pH Homeostasis After Ischemia Reperfusion Injury by CEST‐MRI,” NMR in Biomedicine 30, no. 7 (2017): 1–7, 10.1002/nbm.3720. 28370530

[12] D. L. Longo , A. Busato , S. Lanzardo , F. Antico , and S. Aime , “Imaging the pH Evolution of an Acute Kidney Injury Model by Means of Iopamidol, a MRI‐CEST pH‐Responsive Contrast Agent: MRI‐CEST pH Evolution in an AKI Mouse Model,” Magnetic Resonance in Medicine 70, no. 3 (2012): 859–864, 10.1002/mrm.24513. 23059893

[13] P. Rajkumar and J. L. Pluznick , “Acid‐Base Regulation in the Renal Proximal Tubules: Using Novel pH Sensors to Maintain Homeostasis,” American Journal of Physiology. Renal Physiology 315, no. 5 (2018): F1187–F1190, 10.1152/ajprenal.00185.2018. 30066586 PMC6293293

[14] J. Tostain , G. Li , A. Gentil‐Perret , and M. Gigante , “Carbonic Anhydrase 9 in Clear Cell Renal Cell Carcinoma: A Marker for Diagnosis, Prognosis and Treatment,” European Journal of Cancer 46, no. 18 (2010): 3141–3148, 10.1016/j.ejca.2010.07.020. 20709527

[15] Y. Xu , Q. Wan , X. Ren , et al., “Amide Proton Transfer‐Weighted MRI for Renal Tumors: Comparison With Diffusion‐Weighted Imaging,” Magnetic Resonance Imaging 106 (2024): 104–109, 10.1016/j.mri.2023.12.002. 38135260

[16] M. Zaiss , K. Herz , A. Deshmane , et al., “Possible Artifacts in Dynamic CEST MRI due to Motion and Field Alterations,” Journal of Magnetic Resonance 298 (2019): 16–22, 10.1016/j.jmr.2018.11.002. 30500568

[17] K. Jones , C. Stuehm , C. Hsu , P. Kuo , M. Pagel , and E. Randtke , “Imaging Lung Cancer by Using Chemical Exchange Saturation Transfer MRI With Retrospective Respiration Gating,” Tomography 3, no. 4 (2017): 201–210, 10.18383/j.tom.2017.00017. 29479563 PMC5823523

[18] Z. Chen , C. Liu , Y. Wang , et al., “Free‐Breathing Abdominal Chemical Exchange Saturation Transfer Imaging Using Water Presaturation and Respiratory Gating at 3.0 T,” NMR in Biomedicine 37, no. 8 (2024): e5134, 10.1002/nbm.5134. 38459747

[19] P. M. Robson , A. J. Madhuranthakam , W. Dai , I. Pedrosa , N. M. Rofsky , and D. C. Alsop , “Strategies for Reducing Respiratory Motion Artifacts in Renal Perfusion Imaging With Arterial Spin Labeling,” Magnetic Resonance in Medicine 61, no. 6 (2009): 1374–1387, 10.1002/mrm.21960. 19319891 PMC2946256

[20] J. Keupp , X. Wang , I. E. Dimitrov , H. Eggers , and E. Vinogradov , “Fat‐Corrected APTw/CEST‐MRI with Timed Breathing for Renal Applications,” in *Proceedings of the ISMRM & ISMRT Annual Meeting & Exhibition*, Hawaii, 2025.

[21] X. Wang , I. Dimitrov , J. Keupp , et al., “Accelerating CEST‐MRI for 3D Renal Imaging,” in *Proceedings of the ISMRM & ISMRT Annual Meeting & Exhibition*, 2024, 10.58530/2024/4460.

[22] S. Zhang , B. Li , and J. Greer , “Toward CEST MRI of renal masses: protocol optimization and first preliminary data,” in *Proceedings of the Joint Annual Meeting ISMRM‐ESMRMB*, 2018, https://archive.ismrm.org/2018/5108.html.

[23] X. Wang , Y. Y. Cao , Y. Jiang , et al., “Effects of Breathing Patterns on Amide Proton Transfer MRI in the Kidney: A Preliminary Comparative Study in Healthy Volunteers and Patients With Tumors,” Journal of Magnetic Resonance Imaging 60, no. 1 (2024): 222–230, 10.1002/jmri.29099. 37888865

[24] X. Wang , J. Keupp , I. E. Dimitrov , et al., “Evaluation of Renal Masses With CEST: Protocol Optimization and Preliminary Results,” Magnetic Resonance in Medicine 94, no. 6 (2025): 2374–2387, 10.1002/mrm.30641. 40693363 PMC12501682

[25] E. Vinogradov , Z. Liu , A. J. Madhuranthakam , et al., “Endogenous Urea CEST (urCEST) for MRI Monitoring of Kidney Function,” in *Proceedings of the 23rd Annual Meeting and Exhibition of ISMRM*, 2015.

[26] J. Stabińska , “Development of Quantitative Chemical Exchange Saturation Transfer MRI for Functional Kidney Imaging,” 2020, https://docserv.uni‐duesseldorf.de/servlets/DocumentServlet?id=54463.

[27] X. Xu , A. A. Sehgal , N. N. Yadav , et al., “ d‐glucose Weighted Chemical Exchange Saturation Transfer (glucoCEST)‐based Dynamic Glucose Enhanced (DGE) MRI at 3T: Early Experience in Healthy Volunteers and Brain Tumor Patients,” Magnetic Resonance in Medicine 84, no. 1 (2020): 247–262, 10.1002/mrm.28124. 31872916 PMC7083699

[28] World Health Organization , “Indicator Metadata Registry: Mean Fasting Blood Glucose,” 2024, https://www.who.int/data/gho/indicator‐metadata‐registry/imr‐details/2380.

[29] A. K. Dash , Y. Mo , and A. Pyne , “Solid‐State Properties of Creatine Monohydrate,” Journal of Pharmaceutical Sciences 91, no. 3 (2002): 708–718, 10.1002/jps.10073. 11920756

[30] A. P. Dagher , A. Aletras , P. Choyke , and R. S. Balaban , “Imaging of Urea Using Chemical Exchange‐Dependent Saturation Transfer at 1.5T,” Journal of Magnetic Resonance Imaging 12, no. 5 (2000): 745–748, 10.1002/1522-2586(200011)12:5<745::AID-JMRI12>3.0.CO;2-H. 11050645

[31] Y. Wu , I. Y. Zhou , T. Igarashi , D. L. Longo , S. Aime , and P. Z. Sun , “A Generalized Ratiometric Chemical Exchange Saturation Transfer (CEST) MRI Approach for Mapping Renal pH Using Iopamidol,” Magnetic Resonance in Medicine 79, no. 3 (2017): 1553–1558, 10.1002/mrm.26817. 28686805 PMC5756701

[32] R. Song , A. Tipirneni , P. Johnson , R. B. Loeffler , and C. M. Hillenbrand , “Evaluation of Respiratory Liver and Kidney Movements for MRI Navigator Gating,” Journal of Magnetic Resonance Imaging 33, no. 1 (2011): 143–148, 10.1002/jmri.22418. 21182132 PMC4539151

[33] D. Pham , T. Kron , F. Foroudi , M. Schneider , and S. Siva , “A Review of Kidney Motion Under Free, Deep and Forced‐Shallow Breathing Conditions: Implications for Stereotactic Ablative Body Radiotherapy Treatment,” Technology in Cancer Research & Treatment 13, no. 4 (2014): 315–323, 10.7785/tcrt.2012.500387. 24325129

[34] M. A. Russo , D. M. Santarelli , and D. O'Rourke , “The Physiological Effects of Slow Breathing in the Healthy Human,” Breathe 13, no. 4 (2017): 298–309, 10.1183/20734735.009817. 29209423 PMC5709795

[35] W. M. Liang , Y. X. Ji , J. Xiao , et al., “Respiratory Patterns and Physical Fitness in Healthy Adults: A Cross‐Sectional Study,” BMC Public Health 24, no. 1 (2024): 228, 10.1186/s12889-024-17687-8. 38243241 PMC10797802

[36] P. Virtanen , R. Gommers , T. E. Oliphant , et al., “SciPy 1.0: Fundamental Algorithms for Scientific Computing in Python,” Nature Methods 17, no. 3 (2020): 261–272, 10.1038/s41592-019-0686-2. 32015543 PMC7056644

[37] C. R. Harris , K. J. Millman , S. J. van der Walt , et al., “Array Programming With NumPy,” Nature 585, no. 7825 (2020): 357–362, 10.1038/s41586-020-2649-2. 32939066 PMC7759461

[38] A. Ross , “fmri‐physio‐log: Parse Siemens PMU Files,” 2024, https://github.com/andrewrosss/fmri‐physio‐log.

[39] P. A. Yushkevich , J. Piven , H. C. Hazlett , et al., “User‐Guided 3D Active Contour Segmentation of Anatomical Structures: Significantly Improved Efficiency and Reliability,” NeuroImage 31, no. 3 (2006): 1116–1128, 10.1016/j.neuroimage.2006.01.015. 16545965

[40] H. J. Wittsack , K. L. Radke , J. Stabinska , A. Ljimani , and A. Müller‐Lutz , “Calf – Software for CEST Analysis With Lorentzian Fitting,” Journal of Medical Systems 47, no. 1 (2023): 39, 10.1007/s10916-023-01931-6. 36961580 PMC10038975

[41] F. Charlier , M. Weber , S. Proost , et al., “Statannotations,” November 2024, 10.5281/zenodo.14258156.

[42] C. Ariyurek , T. E. Wallace , T. Kober , S. Kurugol , and O. Afacan , “Prospective Motion Correction in Kidney MRI Using FID Navigators,” Magnetic Resonance in Medicine 89, no. 1 (2023): 276–285, 10.1002/mrm.29424. 36063497 PMC9670860

[43] H. Song , D. Ruan , W. Liu , et al., “Respiratory Motion Prediction and Prospective Correction for Free‐Breathing Arterial Spin‐Labeled Perfusion MRI of the Kidneys,” Medical Physics 44, no. 3 (2017): 962–973, 10.1002/mp.12099. 28074528 PMC5474101

[44] K. Zhang , S. M. F. Triphan , C. H. Ziener , et al., “Navigator‐Based Slice Tracking for Kidney pCASL Using Spin‐Echo EPI Acquisition,” Magnetic Resonance in Medicine 90, no. 1 (2023): 231–239, 10.1002/mrm.29621. 36806110

[45] B. Li , H. She , S. Zhang , et al., “Image Registration With Structuralized Mutual Information: Application to CEST,” in *Proceedings of the 25th Annual Meeting of the International Society for Magnetic Resonance in Medicine (ISMRM)*, Honolulu, HI, USA, 2017, 1293, https://cds.ismrm.org/protected/17MProceedings/PDFfiles/1293.html.

[46] J. Cui , C. Sun , and Z. Zu , “NOE‐Weighted Imaging in Tumors Using Low‐Duty‐Cycle 2π‐CEST,” Magnetic Resonance in Medicine 89, no. 2 (2023): 636–651, 10.1002/mrm.29475. 36198015 PMC9792266

[47] C. K. Jones , A. Huang , J. Xu , et al., “Nuclear Overhauser Enhancement (NOE) Imaging in the Human Brain at 7T,” NeuroImage 77 (2013): 114–124, 10.1016/j.neuroimage.2013.03.047. 23567889 PMC3848060

[48] J. Stabinska , A. Singh , N. M. Haney , et al., “Noninvasive Assessment of Renal Dynamics and pH in a Unilateral Ureter Obstruction Model Using DCE MR‐CEST Urography,” Magnetic Resonance in Medicine 89, no. 1 (2022): 343–355, 10.1002/mrm.29436. 36089805 PMC9753579

[49] J. Wu , Y. Wu , Y. Sun , J. You , W. Zhang , and T. Zhao , “Analysis of Immune Status and Prognostic Model Incorporating Lactate Metabolism and Immune‐Related Genes in Clear Cell Renal Cell Carcinoma,” Discover Oncology 16, no. 1 (2025): 1024, 10.1007/s12672-025-02746-2. 40481935 PMC12145390

[50] K. L. Radke , D. B. Abrar , M. Frenken , et al., “Chemical Exchange Saturation Transfer for Lactate‐Weighted Imaging at 3 T MRI: Comprehensive In Silico, In Vitro, In Situ, and In Vivo Evaluations,” Tomography 8, no. 3 (2022): 1277–1292, 10.3390/tomography8030106. 35645392 PMC9149919

[51] X. Li , X. Xiang , and H. Lin , “Differential Diagnostic Value of Magnetic Resonance Diffusion‐Weighted Imaging and Apparent Diffusion Coefficient for Renal Clear Cell Carcinoma and Non‐Clear Cell Carcinoma,” Oncology Letters 25, no. 2 (2022): 60, 10.3892/ol.2022.13647. 36644151 PMC9827469

[52] P. Z. Sun , “Quasi‐Steady‐State Chemical Exchange Saturation Transfer (QUASS CEST) MRI Analysis Enables *T* _1_ Normalized CEST Quantification—Insight Into *T* _1_ Contribution to CEST Measurement,” Journal of Magnetic Resonance 329 (2021): 107022, 10.1016/j.jmr.2021.107022. 34144360 PMC8316384

[53] M. Wolf , A. de Boer , K. Sharma , et al., “Magnetic Resonance Imaging *T* _1_‐ and *T* _2_‐Mapping to Assess Renal Structure and Function: A Systematic Review and Statement Paper,” Nephrology, Dialysis, Transplantation 33, no. suppl_2 (2018): ii41–ii50, 10.1093/ndt/gfy198. PMC6106643 30137583

[54] K. L. Radke , B. Kamp , V. Adriaenssens , et al., “Deep Learning‐Based Denoising of CEST MR Data: A Feasibility Study on Applying Synthetic Phantoms in Medical Imaging,” Diagnostics 13, no. 21 (2023): 3326, 10.3390/diagnostics13213326. 37958222 PMC10650582

[55] T. Quan , Z. Chen , Y. Zhang , W. Zhang , Y. Xu , and Y. Feng , “Renal 4D CEST‐MRI Under Free Breathing,” in International Conference on Future of Medicine and Biological Information Engineering (MBIE 2024), ed. Y. Yao , X. Li , and X. Yu (SPIE, 2024), 51, 10.1117/12.3048046.

[56] X. Xu , R. Leforestier , D. Xia , K. T. Block , and L. Feng , “MRI of GlycoNOE in the Human Liver Using GraspNOE‐Dixon,” Magnetic Resonance in Medicine 93, no. 2 (2025): 507–518, 10.1002/mrm.30270. 39367632 PMC12050115

[57] A. Deshmane , M. Zaiss , T. Lindig , et al., “3D Gradient Echo Snapshot CEST MRI With Low Power Saturation for Human Studies at 3T,” Magnetic Resonance in Medicine 81, no. 4 (2019): 2412–2423, 10.1002/mrm.27569. 30431179 PMC6718050

[58] M. Sedykh , P. Liebig , K. Herz , et al., “Snapshot CEST++: Advancing Rapid Whole‐Brain APTw‐CEST MRI at 3 T,” NMR in Biomedicine 36, no. 10 (2023): 1–7, 10.1002/nbm.4955. 37076984

