# Supplementary material for: Investigation of Endogenous Renal CEST Contrast and the Influence of Respiratory Motion on a Clinical 3 Tesla MRI: An In Vivo and In Vitro Study
Source: Magn Reson Med. 2025 Dec 3;95(4):2194–206. doi: 10.1002/mrm.70210 (PMC12850607; doi:10.1002/mrm.70210)
Supplement: Supplementary file 2 — Supplemental Digital Content 2. sampling_animation.html. The HTML file is an interactive animation illustrating image acquisition during a CEST scan along a regular sinusoidal breathing curve. [file MRM-95-2194-s001.html]

# Investigation of Endogenous Renal CEST Contrast and the Influence of Respiratory Motion on a Clinical 3 Tesla MRI: An in vivo and in vitro study.

MS 1Patrik Jan Gallinnis, PhD 1\*Benedikt Kamp, MS 1Karl Ludger Radke, MS 1Rika Möller, MS 1Anna-Katharina Juric, PhD 2,3Julia Stabinska, PhD 4Vít Herynek, MD 1,5Gerald Antoch, PhD 1Hans-Jörg Wittsack, MD 1Alexandra Ljimani, PhD 1Anja Müller-Lutz

1Department of Diagnostic and Interventional Radiology Medical Faculty and University Hospital Düsseldorf, Heinrich-Heine-University Düsseldorf, Düsseldorf Germany; 2F.M. Kirby Research Center for Functional Brain Imaging, Kennedy Krieger Institute, Baltimore, Maryland, USA; 3Russell H. Morgan Department of Radiology and Radiological Science, Johns Hopkins University School of Medicine, Baltimore, Maryland, USA; 4Center for Advanced Preclinical Imaging (CAPI), First Faculty of Medicine, Charles University, 120 00 Prague, Czech Republic; 5CARID Cardiovascular Research Institute Düsseldorf, University Hospital Düsseldorf, Heinrich-Heine-University Düsseldorf Germany.


Breathing Rate (BPM):

Sampling Frequency (Images/min):

PPM Range (±):  ppm

Number of Samples (N):
